# *OsMBF1a* Facilitates Seed Germination by Regulating Biosynthesis of Gibberellic Acid and Abscisic Acid in Rice

**DOI:** 10.3390/ijms25189762

**Published:** 2024-09-10

**Authors:** Xin Wang, Ziyun Chen, Jinghua Guo, Xiao Han, Xujian Ji, Meicheng Ke, Feng Yu, Pingfang Yang

**Affiliations:** State Key Laboratory of Biocatalysis and Enzyme Engineering, School of Life Sciences, Hubei University, Wuhan 430062, China; wangxin028@163.com (X.W.); yuknmaistriey@163.com (Z.C.); 17884809214@163.com (J.G.); 202221107011760@stu.hubu.edu.cn (X.H.); jixujian2021@163.com (X.J.); 202031107010172@stu.hubu.edu.cn (M.K.)

**Keywords:** rice, *OsMBF1a*, seed germination, abscisic acid, gibberellin acid

## Abstract

Seed germination is a pivotal stage in the plant life cycle, orchestrated by a myriad of internal and external factors, notably plant hormones. The underlying molecular mechanisms governing rice seed germination remain largely unelucidated. Herein, we uncover *OsMBF1a* as a crucial regulatory factor that employs a dual strategy to promote seed germination: positively activating genes involved in gibberellin (GA) biosynthesis pathways, while negatively regulating key genes responsible for abscisic acid (ABA) synthesis. Furthermore, *OsMBF1a* modulates the endogenous levels of ABA and GA in rice seeds, reinforcing its central role in the germination process. The expression of *ZmMBF1a* and *ZmMBF1b*, the homologous genes in maize, in rice seeds similarly affects germination, indicating the conserved functionality of MBF1 family genes in regulating seed germination. This study provides novel insights into the molecular mechanisms underlying rice seed germination and underscores the significance of MBF1 family genes in plant growth and development.

## 1. Introduction

As a fundamental component of global agriculture, rice is essential for ensuring food security and nutrition around the world. It can adapt to diverse environments, ranging from flooded rice paddies to arid soil, due to the intricate regulatory networks within its cells that control the expression of genes critical for stress tolerance and nutrient absorption. Successful germination ensures the uniform and timely emergence of seedlings, which is vital for optimal plant growth and development. Proper germination influences the plant’s ability to withstand environmental stresses, absorb nutrients efficiently, and achieve high productivity. Consequently, understanding and improving the factors that affect rice seed germination can significantly boost agricultural outcomes and contribute to global food security [1,2]. 

Seed germination is a complex reprogramming process that involves breaking dormancy and undergoing significant biomechanical changes [3]. A crucial aspect of this process is the balance between abscisic acid (ABA) and gibberellic acid (GA), where ABA functions as a negative regulator and GA as a positive one [4,5,6]. In Arabidopsis seeds, the expression of the *9-cis-epoxycarotenoid dioxygenase (NCED)* gene increases ABA levels, inhibiting germination [7,8]. The ABA-responsive transcription factor MYB96 further mediates ABA biosynthesis through *NCED2* and *NCED6*, impacting seed germination [9,10]. This highlights the role of the ABA biosynthesis genes and their regulators in controlling seed germination. ABA operates through the PYR/PYL/RCAR-PP2C-SnRKs signaling cascade [11], with downstream components such as *ABI1*, *ABI2*, *ABI3*, and *ABI5* acting as key regulators in seed germination [4]. Recent research has identified additional components, including the SnRK2.3/SRK2I kinase and protein phosphatase *OsPP2C51*, that positively regulate seed germination [12,13]. On the other hand, GA antagonizes ABA during germination, maintaining high levels to suppress ABA-triggered signaling. Genes like *OsLOL1* and *Seed Dormancy1-2*, which regulate GA biosynthesis, also influence germination [14,15]. Both GA and ABA are synthesized from the common precursor geranylgeranyl diphosphate (GGPP). Understanding the regulators that balance GA and ABA biosynthesis is essential for modulating seed germination effectively.

The Multiprotein Bridging Factor 1 (MBF1) family is an ancient superfamily in all eukaryotic genomes. It contains conserved N-terminal and C-terminal helix-turn-helix domains [16]. This family is classified into two groups: Group I, which includes MBF1a and MBF1b, and Group II, which includes *MBF1c* [17]. In plants, MBF1 proteins are involved in various developmental and stress processes, with a significant focus on their role in stress responses. Among the members of the MBF1 family, the role of *MBF1c* in response to heat stress has received the most extensive research attention and scientific examination. The expression of MBF1c in Arabidopsis and rice enhances heat tolerance, by interacting with TPS5 to modulate trehalose accumulation [18,19,20]. A potato Group I MBF1 gene is inducible by H_2_O_2_ treatment [21], and overexpression lines of grape Group I MBF1 in Arabidopsis result in the reduced accumulation of O_2_- and H_2_O_2_ [22], indicating a potential function in oxidative stress. Additionally, plant MBF1s are involved in responses to osmotic, salinity, drought, and cold effects [16]. However, few studies have focused on the roles of MBF1 in plant development. Overexpressing *MBF1c* in Arabidopsis leads to early flowering and an increased number of seeds, while overexpressing *MBF1c* in soybean enhanced yield [19,23]. Loss-of-function mutations in MBF1 affect development; for example, tomato seeds with MBF1 mutations exhibit an altered germination phenotype [24], and Arabidopsis MBF1 triple mutants showed lower germination rates and higher ABA levels after imbibition [25]. Notably, Arabidopsis *MBF1c* acts as a transcriptional regulator that binds the CTAGA DNA element to regulate the expression of heat-responsive genes [23], highlighting its essential role in plant stress responses and development. 

Rice serves as a model crop plant for investigating functional genomics, and two MBF1 members, *OsMBF1a* and *OsMBF1c*, have been identified in its genome [26]. However, their roles in the development processes, especially in seed germination and seedling stage that are critical for rice growth and yield, are not well understood. *OsMBF1a* has highly expression levels in various tissues, implying its potential functions in rice. To elucidate the roles of *OsMBF1a*, overexpressing lines and CRISPR/Cas9-mediated mutants were created. Phenotypic investigations, transcriptomic sequencing, and hormone quantification revealed that *OsMBF1a* regulates seed germination by modulating the biosynthesis of ABA and GA. Furthermore, the expression of *ZmMBF1a* and *ZmMBF1b*, the closest homologs in maize, in rice also demonstrated their roles in seed germination, suggesting conserved functions of Group I MBF1 in cereal crops. Our findings enrich the understanding of the complex regulatory networks governing seed germination and may facilitate the development of novel strategies for rice breeding programs.

## 2. Results

### 2.1. OsMBF1a Is Ubiquitously Expressed in Rice and Is Induced during Seed Germination

**Figure 1:**
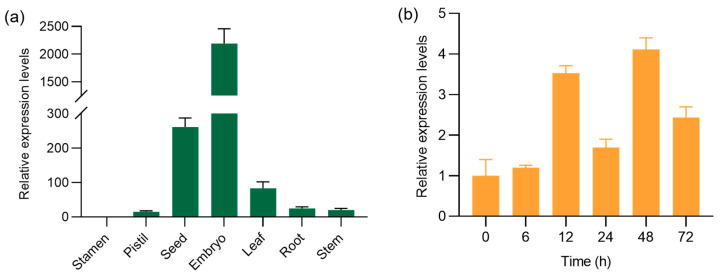
*OsMBF1a* expression profiles in rice tissues and during seed germination. (**a**) Relative expression levels of the *OsMBF1a* gene in various tissues of Nipponbare (NIP) rice, including stamen, pistil, seed, embryo, leaf, root, and stem, as determined by real-time quantitative reverse transcript PCR (RT-qPCR). (**b**) Expression dynamics of *OsMBF1a* throughout the germination process of NIP seeds, assessed using RT-qPCR. Data are presented as mean ± standard deviation (SD) from three biological replicates.

To characterize the expression of *OsMBF1a* in rice, expression data from developmental tissues of Nipponbare (NIP) were extracted from the Bio-Analytic Resource (https://bar.utoronto.ca/eplant_rice/, accessed on 22 March 2023, Figure S1a) and The Rice Annotation Project Database (https://rapdb.dna.affrc.go.jp/, accessed on 22 March 2023, Figure S1b). The data revealed that *OsMBF1a* is ubiquitously expressed in various tissues, including leaf, root, stem, inflorescence, anther, pistil, embryo, and endosperm, with the highest expression levels observed in seed, leaf, and inflorescence. To validate these findings, a real-time quantitative reverse transcript PCR (RT-qPCR) was conducted on the NIP stamen, pistil, seed, embryo, and seedling’s leaf, root, and stem. These results confirmed that the highest expression levels were in the embryo and seed (Figure 1a). Given the high expression in seeds, we further analyzed the expression profile of *OsMBF1a* during seed germination. The results showed that *OsMBF1a* expression increased to more than 3.5-fold after 12 h of germination and remained at elevated levels until 3 days of germination, although it fluctuated at 24 h (Figure 1b), indicating that *OsMBF1a* is induced during seed germination. 

### 2.2. OsMBF1a Positively Regulates Seed Germination in Rice

**Figure 2:**
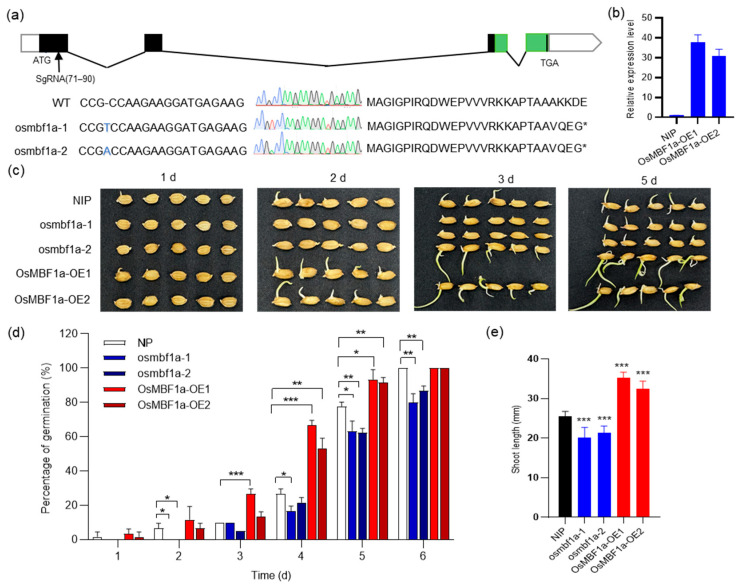
Phenotypic analysis of seed germination in *OsMBF1a* transgenic rice lines. (**a**) Sequences of *OsMBF1a* mutants under the Nipponbare (NIP) rice background, including wild-type (WT) NIP and two mutants, *OsMBF1a*-1 and *OsMBF1a*-2, with mutations in the first exon, chromatogram, and amino acid sequences. In the gene map, the green squares represent domains. The CRISPR/Cas9 target sites are represented by arrows. In the chromatogram, the red curve represents base T, the green curve represents base A, the blue curve represents base C, and the black curve represents base G. (**b**) Relative expression levels of *OsMBF1a* in NIP and two independent overexpression lines, as measured by RT-qPCR. (**c**) Morphological comparison of germinated seeds for NIP and transgenic lines over 1, 2, 3, and 5 day post-germination. (**d**) Percentage germination rate of NIP and transgenic lines over a six-day period. (**e**) Shoot length of NIP and transgenic lines after six days of germination. Data are presented as mean ± standard deviation (SD) from three biological replicates. Statistical significance was determined using a *t*-test. * indicates *p* < 0.05, ** indicates *p* < 0.01, and *** indicates *p* < 0.001.

To delve deeper into the function of *OsMBF1a* in seed germination, we utilized CRISPR/Cas9 technology to mutate the *OsMBF1a* gene within the NIP genetic background. This resulted in two distinct mutant lines: *osmbf1a*-1, which has an extra T at base pair 71 bp of the first exon, and *osmbf1a*-2, with an additional A at the identical location of the *OsMBF1a* gene (Figure 2a). Both mutations led to the premature termination of amino acids, resulting in the deletion of *OsMBF1a*’s functional domains (Figure 2a). Concurrently, plants overexpressing *OsMBF1a* were developed through Agrobacterium-mediated genetic transformation in the NIP background. RT-qPCR analysis confirmed that 11 overexpression lines had markedly elevated levels of *OsMBF1a* transcripts, with the lines *OsMBF1a*-OE1 and *OsMBF1a*-OE2 demonstrating over a 30-fold increase (Figure 2b), indicating successful gene integration and robust transcription. We then evaluated the phenotypes of seed length, seed width, 100-grain weight, and plant height in transgenic lines and found no significant difference between transgenic lines and NIP (Figure S2).

We proceeded to examine the phenotypic consequences of *OsMBF1a* knockout or overexpression on seed germination and early seedling growth in the mutants (*osmbf1a*-1 and *osmbf1a*-2), overexpression lines (*OsMBF1a*-OE1 and *OsMBF1a*-OE2), and the NIP control. Our observations over 6 days revealed that the mutant plants exhibited delayed germination compared to NIP, whereas the overexpression plants showed accelerated germination (Figure 2c). Initially, at one and two days, the mutant seeds showed minimal germination in contrast to the overexpression lines and NIP, which had comparable germination rates (Figure 2d). By the fourth and fifth days of germination, the NIP’s germination rates significantly surpassed that of the mutants but lagged behind the overexpression lines (Figure 2d). On the sixth day, a comparative assessment of seedling height revealed that the overexpression plants had grown approximately 35 mm, whereas the mutant plants reached only about 20 mm, significantly shorter than NIP (Figure 2e). Collectively, these findings suggest that the absence of *OsMBF1a* results in slower seed germination, while its overexpression hastens the process, highlighting the gene’s pivotal role in seed germination.

### 2.3. OsMBF1a Modulates Terpenoid-Related Metabolic Pathways

**Figure 3:**
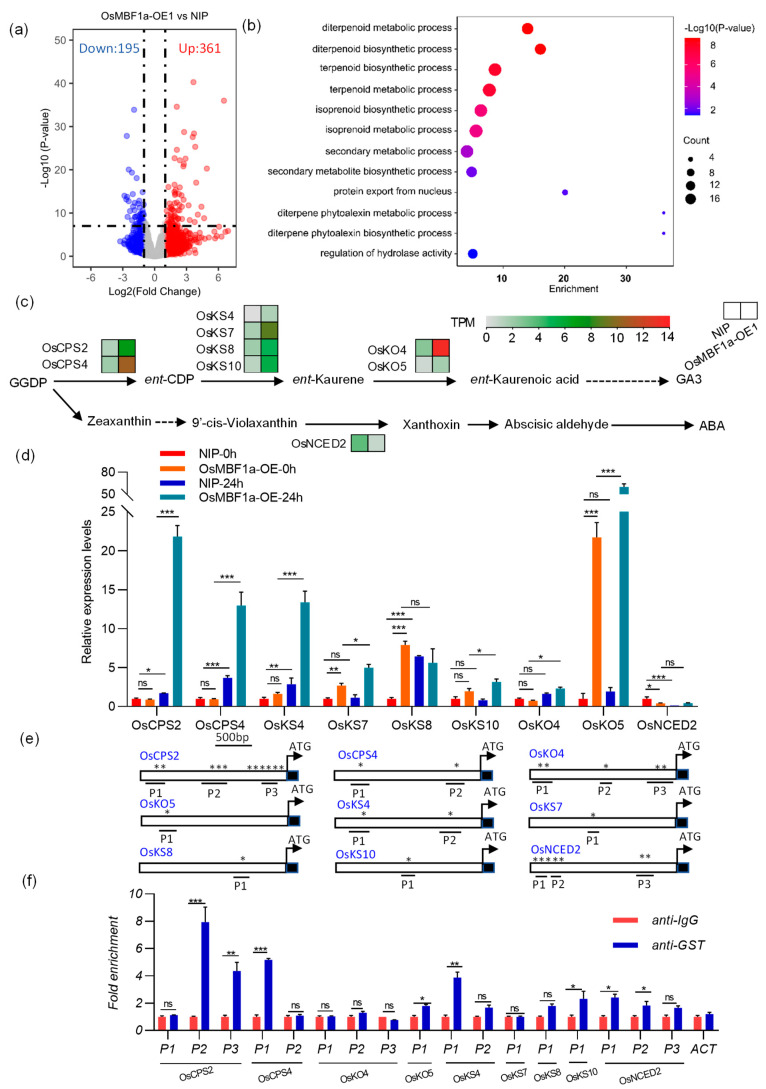
Expression network regulated by *OsMBF1a*. (**a**) Volcano plot illustrating differentially expressed genes (DEGs) in plants overexpressing *OsMBF1a*. (**b**) Gene Ontology analysis highlighting the enriched biological processes among the DEGs. (**c**) Schematic representation of the biosynthesis pathway of gibberellin (GA) and abscisic acid (ABA) from geranylgeranyl pyrophosphate (GGPP), with gene expression levels depicted in a heatmap derived from transcriptome data. (**d**) Expression levels of genes involved in GA and ABA biosynthesis in germinated seeds (0 and 24 h) of *OsMBF1a* overexpression plants compared to NIP, as determined by transcriptome analysis. (**e**) Schemes showed the genomic regions of the CTAGA motif of GA and ABA biosynthesis genes in DAP-qPCR. Stars represent the potential binding motifs of *OsMBF1a*. (**f**) DAP-qPCR analysis of OsMBF1a association with the promoters of the tested genes. The fold enrichment was calculated as bound/input by normalization with IgG control set as 1. *Actin* was used as a negative control. The experiments were repeated three times independently, with similar results. Data are presented as mean ± standard deviation (SD) from three biological replicates. Statistical significance was determined using a *t*-test. * indicates *p* < 0.05, ** indicates *p* < 0.01, and *** indicates *p* < 0.001. NIP, Nipponbare. ACT, actin, ns denotes no significant difference.

To explore the gene expression network orchestrated by *OsMBF1a*, we grew transgenic plants (*OsMBF1a*-OE1) alongside NIP at a stable 28 °C for 14 days and then harvested leaf samples. These samples were flash-frozen and pulverized to a fine powder using liquid nitrogen, in preparation for transcriptome sequencing. This analysis uncovered a total of 546 differentially expressed genes (DEGs) when compared to NIP, with 361 genes showing increased expression and 195 genes showing decreased expression (Figure 3a, Table S1). These results highlight the profound impact of elevated *OsMBF1a* levels on the expression of downstream genes, implying a complex regulatory network with both direct and indirect effects on transcription. RT-qPCR validation of 21 randomly selected DEGs confirmed the transcriptomic data’s accuracy, showing similar expression patterns to those identified by sequencing (Figure S3).

To decode the functional roles of *OsMBF1a*-induced transcriptome changes, the DEGs were categorized and analyzed for functional enrichment using Gene Ontology (GO) terms via the g:Profiler online tool (https://biit.cs.ut.ee/gprofiler/gost, accessed on 23 November 2020, Figure 3b, Table S2). The DEGs were notably enriched in biological processes related to terpenoid metabolism, specifically diterpenoid biosynthesis, in which 16 DEGs were involved. Regarding molecular functions, there was a significant enrichment in genes associated with terpene synthase activity. A further detailed analysis demonstrated that eight DEGs were linked to GA biosynthesis, while one DEG was linked to ABA biosynthesis (Figure 3c), indicating a direct role of *OsMBF1a* in modulating ABA and GA biosynthesis. Remarkably, all of the eight DEGs, including *OsCPS2* (*Os02g0571100*), *OsCPS4* (*Os04g0178300*), *OsKS4* (*Os04g0179700*), *OsKS7* (*Os02g0570400*), *OsKS8* (*Os11g0474800*), *OsKS10* (*Os12g0491800*), *OsKO4* (*Os06g0569500*), and *OsKO5* (*Os06g0568600*), were up-regulated in overexpressing plants, whereas *OsNCED2* (*Os12g0435200*), which catalyzes ABA biosynthesis, was down-regulated (Figure 3c), suggesting that *OsMBF1a* stimulates GA biosynthesis while suppressing ABA biosynthesis.

To access whether these expression changes extended to mature seeds, we conducted an RT-qPCR on the same set of genes in *OsMBF1a*-OE1 and NIP (Figure 3d). Notably, *OsKS4*, *OsKS7*, *OsKS8*, *OsKS10*, and *OsKO5* were significantly up-regulated in the overexpressing plants, whereas *OsCPS2*, *OsCPS4*, and *OsKO4* showed no significant change in the GA biosynthesis pathway. Additionally, *OsNCED2* was down-regulated in the overexpressing plants. The examination of an *OsMBF1a* mutant line (*osmbf1a*-1) showed a down-regulated expression of *OsCPS2*, *OsCPS4*, *OsKS4*, *OsKO4*, and *OsKO5*, while the other three genes exhibited patterns similar to NIP (Figure S4). These findings collectively indicate that *OsMBF1a* also regulates ABA and GA biosynthesis in rice seeds.

Considering that MBF1 protein can potentially bind to the CTAGA DNA element, we obtained the 2 kb promoter sequence of the GA and ABA biosynthesis genes (Figure 3e). All of the tested genes contain the CTAGA motif, and the *OsCPS2* promoter has the most amount of the motif (11) while *OsKO5*, *OsKS4*, and *OsKS8* have only one motif. We thus designed the primers that covered these motifs to analyze the relative abundance of OsMBF1a associated with the DNA fragments. The GST-OsMBF1a protein was expressed and purified, and anti-GST and IgG were used to separate the bound DNA fragments through DNA affinity purification (DAP). The DNA fragments before purifying were used as the input control, and qPCR was applied to quantify the abundance of test genes. The results demonstrated that the fragments of P2 and P3 in *OsCPS2*, P1 in *OsCPS4*, P1 in *OsKO5*, P1 in *OsKS4*, P1 in *OsKS10*, and P1 and P2 in *OsNCED2* were significantly enriched in anti-GST compared to IgG (Figure 3f), indicating that these promoters are potentially targets for OsMBF1a.

### 2.4. OsMBF1a Regulated Endogenous ABA and GA Levels in Rice Seed

**Figure 4:**
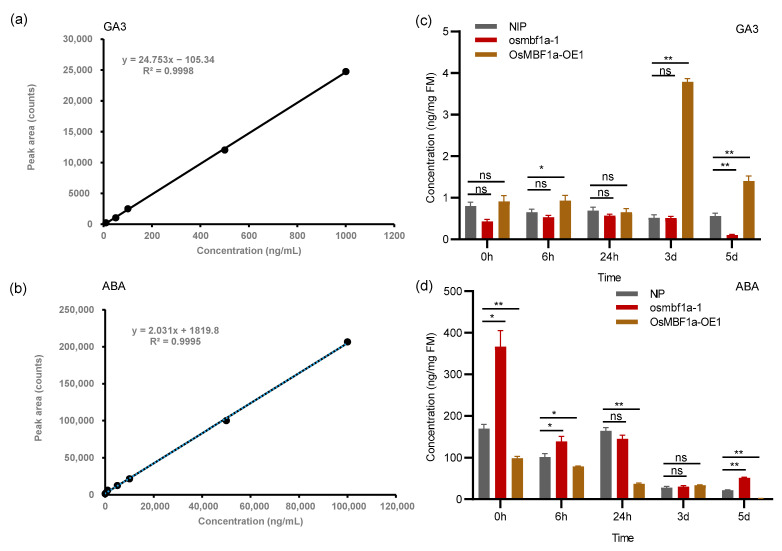
Quantification of ABA and GA content in *OsMBF1a* transgenic rice lines and Nipponbare (NIP). (**a**) Linear regression analysis depicting the relationship between the standard concentration of gibberellin 3 (GA3) and its peak area. (**b**) Linear regression analysis showing the relationship between the standard concentration of abscisic acid (ABA) and its peak area. (**c**) GA3 content measured in seeds of *OsMBF1a* transgenic lines at various germination stages. (**d**) Endogenous ABA content in seeds of *OsMBF1a* transgenic lines at different germination stages. Data are presented as mean ± standard deviation (SD) from three biological replicates. Statistical significance was determined using a *t*-test. * indicates *p* < 0.05, ** indicates *p* < 0.01, and ns indicates no significant.

Given *OsMBF1a*’s involvement in ABA and GA biosynthesis gene expression, we quantified endogenous GA3 and ABA levels in *OsMBF1a*-OE1, *osmbf1a*-1, and NIP seeds at various germination stages using LC-MS/MS. The standard curve-based concentration gradient of GA3 and ABA standard solutions was established by quantification, and the final linear equations of GA3 and ABA were expressed as y = 24.753x − 105.34 for GA3 (R^2^ = 0.9998, Figure 4a) and y = 2.031x + 1819.80 for ABA (R^2^ = 0.9995, Figure 4b). These equations were then utilized to calculate the GA3 and ABA contents in each sample based on their respective peak areas. The GA3 content was modestly elevated in *OsMBF1a*-OE1 and significantly reduced in *osmbf1a*-1 during the initial 0 to 6 h of germination compared with NIP, with more pronounced differences at 3 and 5 days (Figure 4c). After 3 days’ germination, the GA3 content in *OsMBF1a*-OE1 was highest and was approximately 4-fold more than in NIP (Figure 4c). The overexpression of *OsMBF1a* significantly reduced ABA content, whereas the mutant showed increased ABA content in mature seeds, with differences also observed after 6 h of germination (Figure 4d). The highest ABA concentration was in germinated seeds at 0 h, and the content in *osmbf1a*-1 was about 2-fold more than in NIP. ABA levels decreased progressively during germination, with significant differences among *OsMBF1a*-OE1, *osmbf1a*-1, and NIP evident after 5 days (Figure 4d). These results indicate that *OsMBF1a* overexpression inhibits ABA synthesis and enhances GA3 production during seed germination, correlating with the observed germination phenotypes. 

### 2.5. Overexpressing ZmMBF1a and ZmMBF1b in Rice Enhances Seed Germination

**Figure 5:**
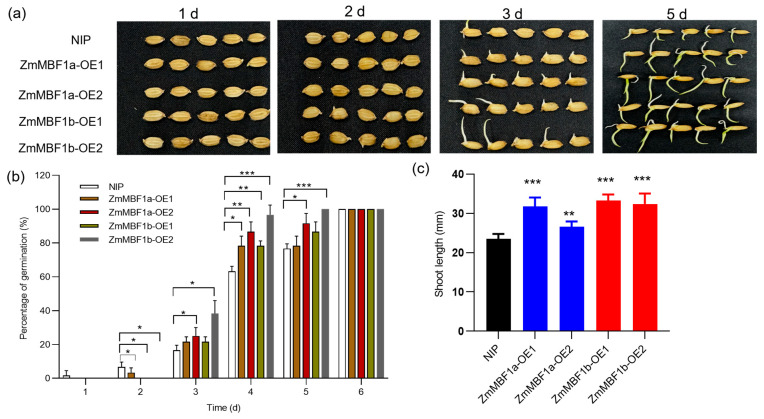
Seed germination phenotypes in *ZmMBF1a* and *ZmMBF1b* transgenic rice lines. (**a**) Comparative images of germinated seeds for NIP (wild-type) and transgenic lines over a period of 1, 2, 3, and 5 days, illustrating the developmental progression post-germination. (**b**) Cumulative germination rate of NIP and transgenic lines, measured daily for a duration of six days, highlighting the temporal dynamics of seed germination. (**c**) Quantitative assessment of shoot length for NIP and transgenic lines at the six-day mark post-germination. Data are presented as mean ± standard deviation (SD) from three biological replicates. Statistical significance was determined using a *t*-test. * indicates *p* < 0.05, ** indicates *p* < 0.01, and *** indicates *p* < 0.001. NIP, Nipponbare; *ZmMBF1a*-OE1 and *ZmMBF1a*-OE2, two independent transgenic lines that overexpressed *ZmMBF1a*; *ZmMBF1b*-OE1 and *ZmMBF1b*-OE2, two independent transgenic lines that overexpressed *ZmMBF1b*.

To determine if *OsMBF1a*’s role in seed germination is conserved across plant species, we identified MBF1 homologs in rice, maize, and Arabidopsis. Phylogenetic analysis revealed that *ZmMBF1a* and *ZmMBF1b* are the closest maize homologs to *OsMBF1a*, with a high protein sequence similarity (Figure S5), suggesting conserved functions. We cloned the full-length coding sequence of *ZmMBF1a* and *ZmMBF1b* from maize inbred line B73 leaves and expressed them constitutively in rice, generating transgenic lines named *ZmMBF1a*-OE and *ZmMBF1b*-OE (Figure S6a). Positive transformation was confirmed in two lines per gene (Figure S6b,c). Compared to NIP, these lines exhibited an enhanced germinating ability (Figure 5a). Growth curves based on seedling height data over 6 days indicated that the overexpression lines surpassed NIP in germination rates after the third day (Figure 5b). On the sixth day, the seedling height reached approximately 30 mm by the sixth day compared to NIP’s 25 mm (Figure 5c), demonstrating that *ZmMBF1a* and *ZmMBF1b* overexpression promotes seed germination and growth.

### 2.6. ZmMBF1a and ZmMBF1b Overexpression Modulates GA and ABA in Rice

**Figure 6:**
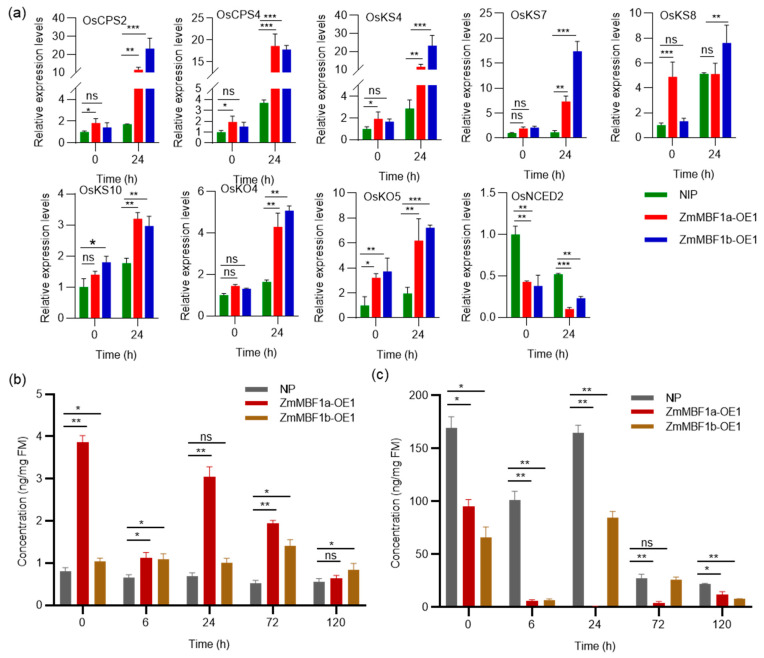
Expression and hormone content analysis in *ZmMBF1a* and *ZmMBF1b* transgenic lines. (**a**) Expression levels of genes involved in GA and ABA biosynthesis in transgenic lines overexpressing *ZmMBF1a* and *ZmMBF1b*. (**b**) Quantitative analysis of GA3 content in seeds of transgenic lines overexpressing *ZmMBF1a* and *ZmMBF1b* during various germination stages. (**c**) Measurement of endogenous ABA content in seeds of the same transgenic lines at different germination stages. Data are presented as mean ± standard deviation (SD) from three biological replicates. Statistical significance was determined using a *t*-test. * indicates *p* < 0.05, ** indicates *p* < 0.01, *** indicates *p* < 0.001, and ns denotes no significant difference.

Considering the germination phenotypes of *ZmMBF1a* and *ZmMBF1b*, we examined the expression of the nine aforementioned genes involved in GA and ABA biosynthesis in NIP, *ZmMBF1a*-OE1, and *ZmMBF1b*-OE1 using RT-qPCR (Figure 6a). The overexpression of *ZmMBF1a* and *ZmMBF1a* slightly increases GA biosynthesis gene expression in transgenic rice seeds, with a significantly higher expression observed 24 h post-germination compared to NIP, indicating that transgenic plants facilitate the expression of GA biosynthesis. A corresponding decrease in *OsNCED2* expression was noted in overexpressing plants. These findings suggest that *ZmMBF1a* and *ZmMBF1b* modulate GA and ABA biosynthesis. An analysis of endogenous ABA and GA content in overexpression lines (*ZmMBF1a*-OE1 and *ZmMBF1b*-OE1) during the first 5 days of germination showed significantly higher GA3 content compared to NIP, except at certain time points (Figure 6b). ABA content decreased significantly within 5 days post-germination, with the most notable decrease 6 h post-germination and the lowest levels in *ZmMBF1a*-OE1 24 h post-germination (Figure 6c). This indicates that *ZmMBF1a* and *ZmMBF1b* overexpression can reduce ABA and increase GA content in rice seeds, potentially enhancing germination. The significant increase in GA3 content and the significant decrease in ABA content in *ZmMBF1a-* and *ZmMBF1b*-overexpressed plants at the early stage of germination further verified the direct effect of changes in gene expression level on hormone levels. It is worth noting that the increase in GA3 content and the decrease in ABA content showed a certain synchronicity in time, especially within 6 to 24 h after germination, which indicates that the regulatory effect of *ZmMBF1a* and *ZmMBF1b* is particularly critical in the early stage of seed germination. In addition, the ABA content of *ZmMBF1a*-OE1 plants reached the lowest level at 24 h, while the GA3 content remained at a high level, which may be one of the reasons for the stronger germination ability of *ZmMBF1a*-OE1 plants.

## 3. Discussion

Seed germination is an intricate and tightly regulated biological process that integrates a variety of physical and biochemical signals. It is determined by a combination of external environmental factors and endogenous genetic mechanisms. Studies have shown that the *OsMFT2* gene, through its interaction with transcription factors *OsbZIP23/66/72*, positively regulates the expression of ABA-responsive genes. This interaction plays a negative regulatory role in seed germination [27]. Additionally, *OsSAE1*, a member of the AP2 family, directly targets the promoter region of *OsABI5*, a key regulatory gene in the ABA signaling pathway. It inhibits *OsABI5*’s expression, accelerating the germination process of rice seeds [28]. Researches have also shown that mutations in the *OsKO1* gene in rice can significantly disrupt the GA biosynthesis pathway. This disruption subsequently affects starch mobilization and ABA signaling, leading to delayed seed germination [29]. This underscores the critical role of hormonal balance in the physiological transition of seeds. Furthermore, the *OsAP2-39* transcription factor effectively maintains the dynamic balance between ABA and GA in rice by finely regulating the expression of the ABA synthesis gene *OsNCED1* and the GA metabolism-related gene *EUI* [30], providing a new perspective on understanding plant hormone interaction networks. These studies imply that a dissection of the regulators involved in GA and ABA metabolism and signaling is an efficient way to modulate seed germination.

In the present study, transgenic rice plants overexpressing *OsMBF1a* exhibited enhanced seed germination, whereas the *OsMBF1a* mutants showed inhibited germination (Figure 2). Expression evidence suggested that *OsMBF1a* modulates the gene expression involved in GA and ABA biosynthesis (Figure 3), leading to increased levels of GA and decreased levels of ABA in transgenic seeds (Figure 4). These findings indicate that *OsMBF1a* is a potential regulator that balances GA and ABA. The HbMBF1a gene enhances ABA insensitivity when overexpressed in Arabidopsis thaliana, indicating its function as a positive regulator of ABA responses [29]. Furthermore, triple knockdown experiments of MBF1 genes in Arabidopsis have illuminated the negative role of MBF1s in ABA-mediated seed germination inhibition, which also suggested potential involvement in ABA signaling regulation [25]. These implied the conserved roles of MBF1 members in regulating ABA-dependent pathway. Given the conserved protein sequence of MBF1a members in rice and maize (Figure S5), we hypothesize conserved functions among them. Overexpression of the homologous genes *ZmMBF1a* and *ZmMBF1b* in rice resulted in enhanced germination ability, increased GA3 levels, and decreased ABA levels (Figure 5 and Figure 6). Moreover, these overexpression plants also affected the gene expression of ABA and GA biosynthesis (Figure 6), similar to transgenic plants of *OsMBF1a*, indicating conserved functions in regulating GA and ABA balance. While previous studies have largely focused on the functions of MBF1 members in stress response, less is known about their roles in plant development [16]. Our results provide direct clues regarding MBF1a’s involvement in plant development. 

The MBF1 family can be categorized into two distinct subgroups; namely, Group I and Group II. It is noteworthy that the majority of research has concentrated on the Group II member, *MBF1c*, particularly in the context of stress responses. For instance, *StMBF1c* has been shown to significantly bolster potato resistance to Ralstonia solanacearum by meticulously modulating the expression of genes associated with the salicylic acid and ABA signaling pathways, as well as through its interaction with *StTPS5* [31]. In a similar vein, TaMBF1c is instrumental in enhancing plant thermotolerance, indicating its substantial potential for improving crop heat resistance [20]. Additionally, the *CsMBF1c* transcription factor in cucumber is known to stabilize the photosynthetic apparatus and augment heat tolerance through specific protein interactions under conditions of elevated temperature [32]. Likewise, the overexpression of *SlER24*, a member of the *MBF1c* subfamily in tomato, markedly improves salt tolerance, underscoring the pivotal role of the MBF1 family in facilitating plant adaptation to a variety of environmental stresses [33]. Conversely, there has been a dearth of research on the Group I members, MBF1a and MBF1b, with regard to their involvement in stress responses. Although *OsMBF1a* is classified as a Group I member of the MBF1 family, the study in question did not delve into its role in stress. However, the conserved functions of MBF1a in rice and maize have been identified in relation to plant development. These findings not only enhance our comprehension of the regulatory mechanisms of MBF1a but also present novel molecular targets for the genetic enhancement of seed germination performance.

## 4. Materials and Methods

### 4.1. Construction and Planting Materials

To create mutant rice plants lacking the *OsMBF1a* gene, we utilized CRISPR/Cas9 technology to perform a gene knockout. A specific single guide RNA (sgRNA) was designed to target the first exon of *OsMBF1a* using an online tool (http://crispr.hzau.edu.cn/CRISPR2/, accessed on 23 November 2020.) and inserted into the XbaI restriction site of the pCXUN vector. For the generation of overexpressing plants for *OsMBF1a*-OE, *ZmMBF1a*-OE, and *ZmMBF1b*-OE in rice, the coding sequences (CDSs) of *OsMBF1a*, *ZmMBF1a,* and *ZmMBF1b* were cloned from cDNA of NIP and maize inbred line B73 leaves, respectively. The successfully cloned CDSs were inserted into the pC1300 backbone vector under the control of ubiquitin promoter using a ClonExpressII One Step Cloning Kit (Vazyme, Nanjing, China), with the CDSs fused to green fluorescent protein. These constructed vectors were then introduced into Nipponbare calli using an Agrobacterium tumefaciens-mediated transformation system. Positive mutant lines were identified through sequencing, and positive overexpressing lines were identified through RT-qPCR, and T2 seeds were used for further analysis. Primer sequences are detailed in Supplementary Table S3. All transgenic plants and NIPs were cultivated at the experimental site in Wuhan, China, and, sampled during the seedling stage for testing. All plants were harvested for T2 seeds at the same time. 

### 4.2. Phenotypic Analysis of Seed Germination

To explore the role of *OsMBF1a* in seed germination and early seedling growth, we cultivated mutants (*osmbf1a-1* and *osmbf1a-2*), overexpression lines of *OsMBF1a* (*OsMBF1a*-OE1 and *OsMBF1a*-OE2), *ZmMBF1a* (*ZmMBF1a*-OE1 and *ZmMBF1a*-OE), and *ZmMBF1b* (*ZmMBF1b*-OE1 and *ZmMBF1b*-OE2), and NIP seeds in Petri dishes. The seeds were completely soaked in water for a duration of 24 h, and then a small amount of water in the Petri dish kept it moist; this time point was set as the starting point (Day 0). All experiments were conducted under the same cultivation conditions, monitoring germination at 1, 2, 3, and 5 days post-cultivation. Seed germination rates were recorded and statistically compared to NIP over a six-day period to access the significance. Each replicate experiment used a total of 20 seeds for germination analysis. The seeds were cultivated in a chamber with a light intensity of 300 μmol·m^−2^·s^−1^, at 28 °C, with a 14 h light/10 h dark cycle and 60% humidity. Seeds from NIP and transgenic lines at different germination time points, with 10 seeds per point, were also collected and stored at −80 °C. These samples were used for subsequent experimental research.

### 4.3. RNA Extraction and RT-qPCR

To investigate the gene expression in germinated seeds, total RNA was isolated using Total RNA Isolation Reagent (Biosharp, Beijing, China). For the expression profiling of *OsMBF1a* in different rice tissues, the seedlings’ root, stem, and leaves, stamen, pistil, and embryo, collecting in our previous study [34], were subjected to quantitative analysis. RNA was reverse-transcribed into cDNA using the HiScript III RT SuperMix for Qpcr (+gDNA wiper) (Vazyme, Nanjing, China). The cDNA was then amplified with specific primers of genes for RT-qPCR using a Bio-Rad CFX384 Real-Time PCR system with SYBR (Table S3). Actin served as the internal control. The relative gene expression was calculated using the 2^−ΔΔCT^ method, and each sample was tested in triplicate for reliability. The PCR involved an initial denaturation step at 95 °C for 5 min, followed by 40 cycles at 95 °C for 10 s, 58 °C for 10 s, and 72 °C for 15 s. 

### 4.4. RNA-Seq Analyses

The *OsMBF1a* overexpression line *OsMBF1a*-OE1 and NIP plants were grown in a 28 °C incubator for 14 days. Leaf samples were collected, ground into powder with liquid nitrogen, and three biological replicates were prepared for RNA sequencing. The raw reads from the Illumina sequencing were quality-controlled, cleaned, and aligned to the rice reference genome IRGSP-1.0. The aligned reads were counted with featureCounts software (version 2.0.6), and gene expression levels were quantified as transcript per million (TPM). A differential expression analysis was performed using the DESeq2 package, identifying DEGs with an adjusted *p*-value < 0.01 and a fold change ≥ 2 or ≤ 0.5. The g:Profiler online tool was used for Gene Ontology (GO) enrichment analysis to elucidate the biological functions, molecular processes, and cellular components of the DEGs.

### 4.5. DAP-qPCR Analyses

The DAP-qPCR was conducted based on previous research [35]. The CDS of *OsMBF1a* cloned from NIP leaves was amplified and inserted into the pGEX-4T-1 vector to generate GST-OsMBF1a fusion protein. The expression of GST-OsMBF1a in *Escherichia coil* Rosetta (DE3) was induced by 0.5 mM isopropyl β-D-thiogalacoside at 16 °C for 16 h. Glutathione Beads 4FF (LABLEAD, Beijing, China) was used to purify the GST-OsMBF1a protein according to the manufacturer’s protocols. The purified protein was incubated with NIP genomic DNA fragments that were sonicated to about 200 to 500 bp using the Ultrasonic Homogenizer JY92-IIN (SCIENTZ, Ningbo, China) to facilitate binding, followed by the enrichment of protein-bound DNA fragments using Anti-GST Beads (Biolinkedin, Shanghai). Rabbit Control IgG (Abclonal, Wuhan, China) was used as a control to enrich other DNA fragments. Using these enriched DNA fragments as templates, primers of the tested genes covering the target motifs were designed for qPCR assays. Primers for DAP-qPCR are listed in Table S3.

### 4.6. Detection of GA3 and ABA Content

Seeds from NIP, the mutant line (*osmbf1a-1*), and overexpression lines (*OsMBF1a*-OE1, *ZmMBF1a*-OE1 and *ZmMBF1b*-OE1) at various germination time points were crushed into powder using liquid nitrogen. A 0.1 g sample was dissolved in 1 mL of 70% methanol water containing 0.05% acetic acid, mixed, and extracted at 4 °C for approximately 12 h at 200 rpm, then centrifuged at 11,000 rpm for 5 min. The supernatant was filtered through a 0.22 µm membrane and analyzed by mass spectrometry. GA3 and ABA standards were prepared in 1 mL of 75% methanol and diluted to create a standard curve. The separation was performed using high-performance liquid chromatography with a 5500+ Qtrap MS system equipped with an electrospray ionization (ESI) source (AB SCIEX, Foster City, CA, USA) and a Waters ACQUITY Premier HSS T3 column (2.1 × 100 mm, 1.8 µm particle size). Data acquisition and processing were conducted using Analyst 1.7.2 software (AB SCIEX, Foster City, CA, USA). The mobile phases consisted of ultrapure water with 0.04% acetic acid (solvent A) and acetonitrile with 0.04% acetic acid (solvent B), with a flow rate of 0.35 mL/min. The gradient program was as follows: 5–95% B, 10.0 min; 95% B, 10.0–13.0 min; 95–5% B, 13.0–13.1 min; and 5% B, 13.1–16.0 min. The column temperature was maintained at 40 °C, and the injection volume was 5 μL. Analytes of GA3 and ABA were detected using the scheduled multiple reaction monitoring (sMRM) mode, and the specific MRM parameters for GA3 and ABA were as referred to in a previous study [36]. The mass spectrometer parameters were set as follows: Electrospray Ionization Source Temperature (ESI), 550 °C; Ion Spray Voltage (IS), −4500 V; Ion Source Gas I (GSI), 60 psi; Ion Source Gas II (GS II), 60 psi; Curtain Gas (CUR), 30 psi.

## Data Availability

The raw reads data of the transcriptome sequencing have been submitted into the Genome Sequence Archive at the National Genomics Data Center, Beijing Institute of Genomics, Chinese Academy of Sciences/China National Center for Bioinformation (GSA: CRA018111), and are publicly accessible at https://ngdc.cncb.ac.cn/gsa/ (accessed on 1 August 2024).

## Supplementary Materials

The following supporting information can be downloaded at: https://www.mdpi.com/article/10.3390/ijms25189762/s1.
