# Supplementary material for: OsMBF1a Facilitates Seed Germination by Regulating Biosynthesis of Gibberellic Acid and Abscisic Acid in Rice
Source: Int J Mol Sci. 2024 Sep 10;25(18):9762. doi: 10.3390/ijms25189762 (PMC11432016; doi:10.3390/ijms25189762)
Supplement: Supplementary file 1 [file ijms-25-09762-s001.zip › ijms-3176497-supplementary.pdf]

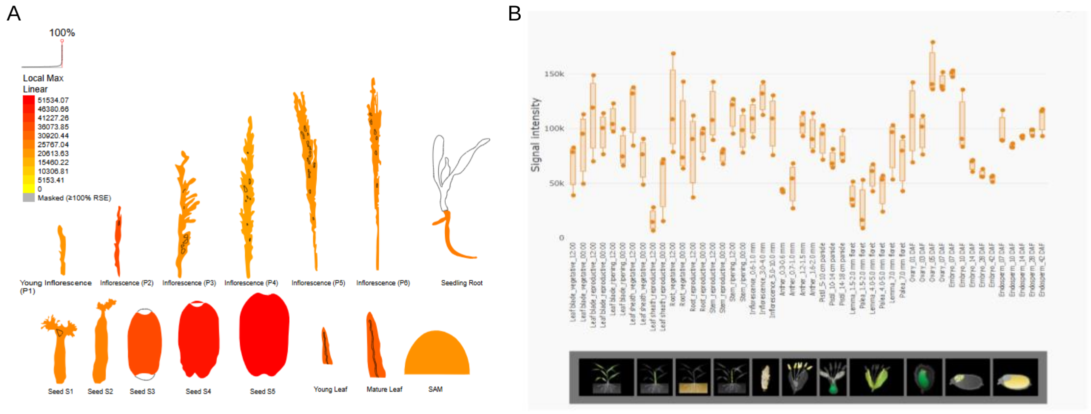

Figure S1. Spatio-temporal expression patterns of *OsMBF1a* in Nipponbare. A) Illustration of the spatio-temporal expression patterns of the *OsMBF1a* gene in Nipponbare as obtained from the Bio-Analytic Resource. B) Presentation of the spatio-temporal expression patterns of *OsMBF1a* in Nipponbare from the Rice Annotation Project Database. DAF indicates days after fertilization.

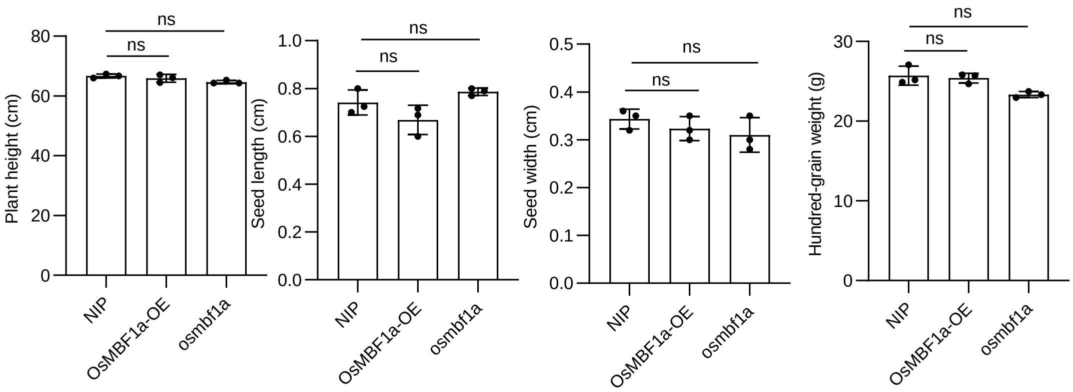

Figure S2. The phenotypes of length, width, and 100-grain weight of seeds and plant height of *OsMBF1a*-OE1 and *osmbf1a*-1. Data are presented as mean ± standard deviation (SD) from three biological replicates. Statistical significance was determined using a t-test. ns, no significant.

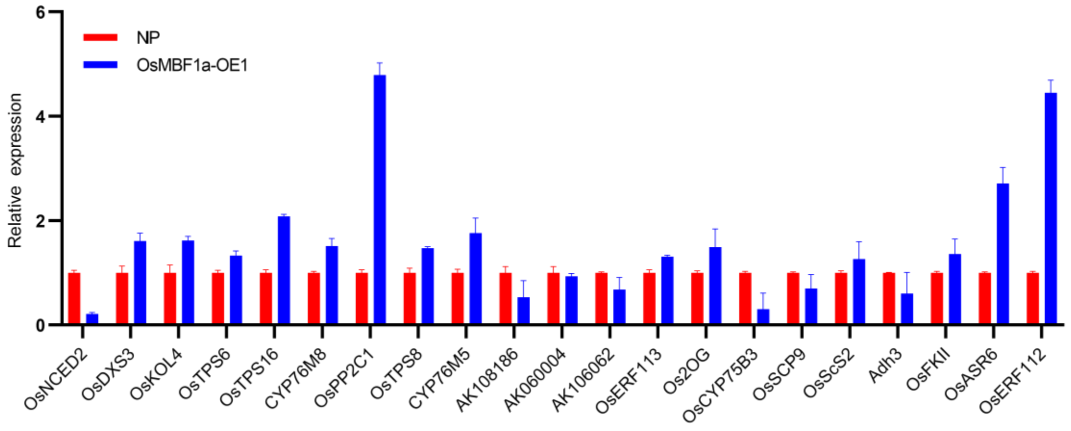

Figure S3. Validation of differential gene expression in *OsMBF1a*-OE1. Validation of differentially expressed genes in the *OsMBF1a* overexpression line (*OsMBF1a*-OE1) compared to the wild-type Nipponbare (NIP) using reverse transcription quantitative PCR (RT-qPCR).

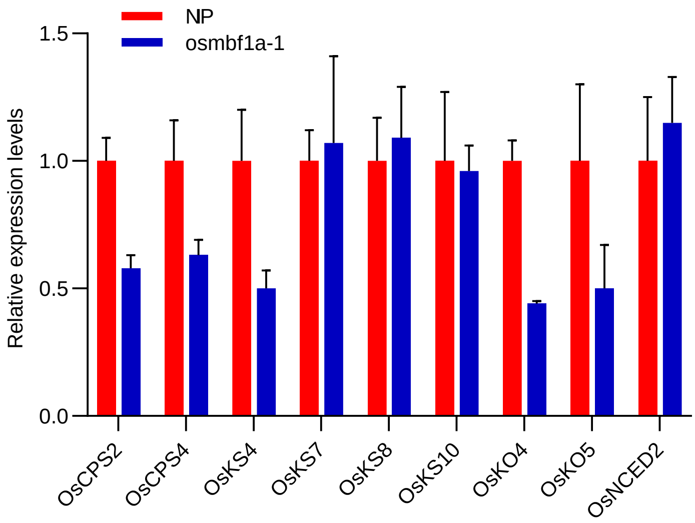

Figure S4. Expression of ABA and GA biosynthesis genes in the osmbfa-1 mutant. Analysis of the expression levels of genes involved in abscisic acid (ABA) and gibberellin (GA) biosynthesis in the osmbfa-1 mutant background.

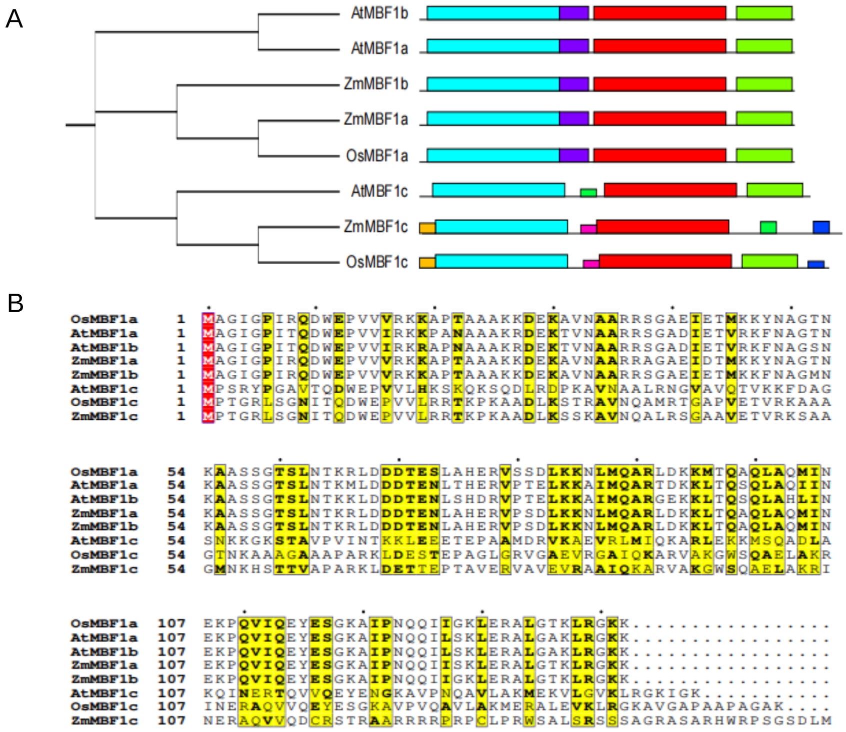

Figure S5. Phylogenetic and sequence analysis of MBF1 family members. A) Examination of the phylogenetic relationship among MBF1 family members in Arabidopsis, rice, and maize. B) Sequence alignment of MBF1 members to highlight conserved and variable regions across species.

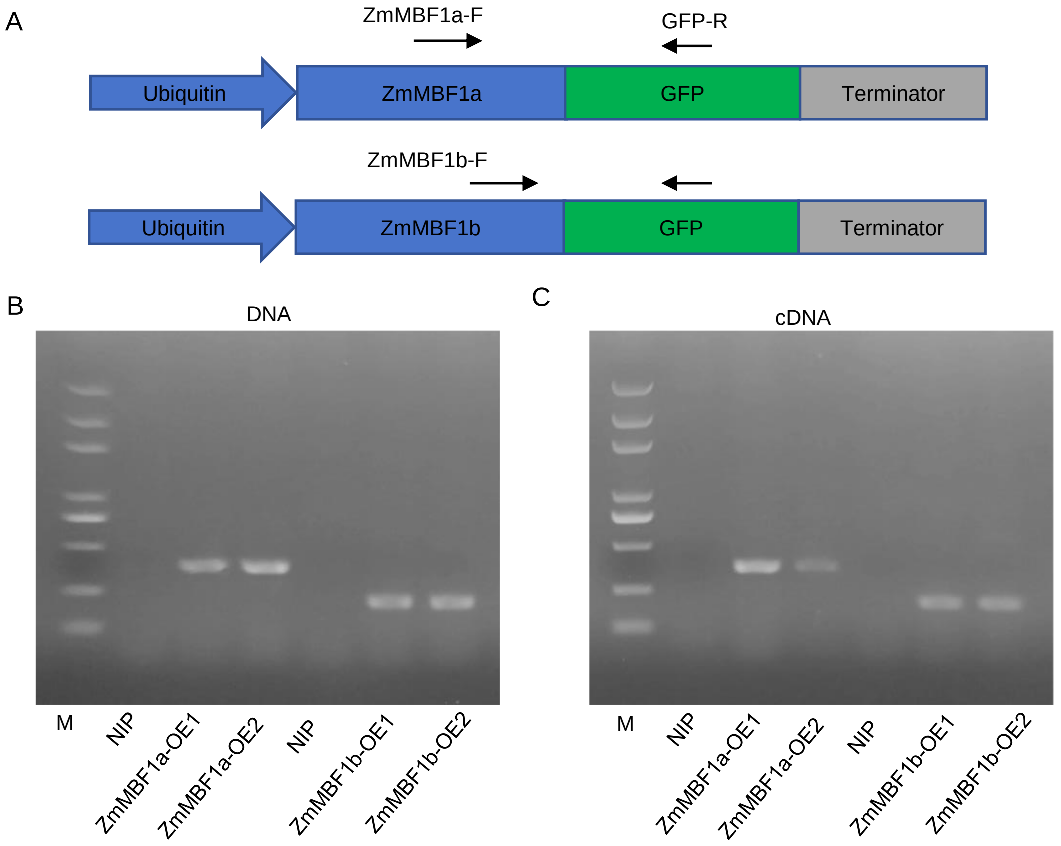

Figure S6. Overexpression of ZmMBF1a and ZmMBF1b in rice. A) Schematic representation of the vector designs used for the overexpression of ZmMBF1a and ZmMBF1b in rice. B) Detection of the insertion of ZmMBF1a and ZmMBF1b in transgenic rice plants using DNA-based methods. C) Analysis of the expression levels of ZmMBF1a and ZmMBF1b in transgenic rice plants using cDNA derived from reverse transcription.
